# Questionnaire-Based Assessment of the Masticatory Function and Facial Nerve Recovery Post Pterional Approach in Brain Tumors Surgery

**DOI:** 10.3390/jcm11010065

**Published:** 2021-12-23

**Authors:** Mihaela Romanița Gligor, Corina Marilena Cristache, Mirela Veronica Bucur, Mihai Burlibasa, Claudiu Matei

**Affiliations:** 1 Department of Dental Medicine and Nursing, Faculty of Medicine, Lucian Blaga University, 2A Lucian Blaga Str., 550169 Sibiu, Romania; romanita.gligor@ulbsibiu.ro; 2 Department of Dental Techniques, Faculty of Midwifery and Medical Assisting (FMAM), Carol Davila University of Medicine and Pharmacy, 8 Eroilor Sanitari Blvd., 050474 Bucharest, Romania; mihai.burlibasa@umfcd.ro; 3 Department of Prosthetic Technology and Dental Materials, Faculty of Dental Medicine, Carol Davila University of Medicine and Pharmacy, 19 Plevnei Ave., 010221 Bucharest, Romania; 4 Department of Neurology, Faculty of Medicine, Lucian Blaga University, 2A Lucian Blaga Str., 550169 Sibiu, Romania; matei.claudiu@ulbsibiu.ro; 5 Department of Neurosurgery, Polisano-Medlife Hospital, 1A Izvorului Str., 550172 Sibiu, Romania

**Keywords:** pterional surgery, masticatory function, patient-centered outcome, atrophy, telemedicine

## Abstract

Background: The pterional approach for craniotomy, one of the most used surgical intervention in neurosurgery, results in a series of postoperative changes that, if they persist, affect the patient’s life, social reintegration, and his/her physical and mental recovery. The aim of the present study was to develop and validate a questionnaire for indicating directly affected masticatory muscles groups and facial nerve branches, in patients undergoing the pterional approach in neurosurgery, so that the recovery therapy can be monitored and personalized. Methods: A self-reporting questionnaire consisting of 18 items (12 for postoperative masticatory status and 6 for facial nerve branches involvement), validated on fifteen patients, following three steps: items development, scale development, and scale evaluation, was prospectively applied twice, at a one-year interval (T0 and T1), with thirty-two patients suffering from vascular or tumoral pathology and surgically treated through a pterional approach. Results: No statistically significant correlation could be found between postoperative outcomes and age or gender. Facial nerve branch involvement could not be correlated with any of the assessed variables. Pathology and time elapsed from surgery were statistically significantly correlated to preauricular pain on the non-operated side (*p* = 0.008 and *p* = 0.034, respectively). Time elapsed from surgery was statistically significantly correlated to the ability to chew hard food, pain while yawning, and preauricular pain during back and forward jaw movements and gradual mouth opening. Conclusions: We created and validated a valuable patient-centered questionnaire that can be employed as a tool for postoperative assessment of directly affected masticatory muscles and groups of facial nerve branches.

## 1. Introduction

The pterional approach for craniotomy is one of the most used surgical interventions in neurosurgery, being recommended for lesions arising along the anterior and middle skull base, such as vascular pathology (aneurysms occurring in the anterior circulation, in the Circle of Willis, or in certain aneurysms in the posterior circulation), anterior and central skull base tumor resections, and also in addressing certain frontal or frontal-temporal intra-axial tumors [1].

Since it was first described by Yasargil et al. in 1976 [2], several improvements of the technique have been made to avoid adverse postoperative changes or complications, such as longer-lasting palsy of frontotemporal facial nerve branches, temporalis muscle atrophy, and temporomandibular joint (TMJ) dysfunction [1].

The pterional surgical intervention applied for brain tumors results in a series of postoperative changes that, if they persist, affect the patient’s life, social reintegration, and his/her physical and mental recovery. The problems identified in this regard are related to post-operative facial asymmetries accompanied by pain in jaw movements, changes in eating habits, pain while eating, and pre-auricular pain [3].

For patients undergoing surgery after vascular pathology or brain tumors, developing a treatment plan to prevent chronic conditions of TMJ and to improve postoperative recovery is mandatory. If a connection between postoperatively affected muscle groups and the surgical technique used could be established [4], a customized protocol, complementary to the one applied for the basic condition, could resolve painful sensitivity to various movements and prevent short- and long-term complications. Thus, there will be a series of immediate positive effects, i.e., attenuation to disappearance of muscle pain, active monitoring of pain in the TMJ, prevention of the installation of joint ankylosis [5], and correction of facial asymmetries.

Due to the fact that patients with functional limitations usually address dental professionals rather than neurosurgeons, for postoperative treatment of masticatory disfunctions or limitations, a multidisciplinary approach will be useful [6].

Following the results of the survey based on the questionnaire, neurosurgeons could find selective solutions for each technique employed to reduce the negative impact of surgery on the TMJ and masticatory muscles. Through interdisciplinary collaboration with a dental practitioner, the directly affected muscle groups can be identified so that the recovery therapy can be personalized. The team will be able to indicate complementary muscle recovery devices and/or specific dental procedures to correct arch or facial asymmetries. The patient will be aware of problems arising as inevitable side effects of surgery and will be able to indicate signs and symptoms that are important in recovery therapy [6].

Patient-reported outcomes acquires directly patient’s feedback regarding their feeling or function in relation to a certain health conditions and are considered by the European Medicines Agency (EMA) and the U.S. Food and Drug Administration (FDA), relevant endpoints for approving new therapies [7]. Thus, well-addressed self-reporting outcomes could improve surgical techniques and postoperative care.

To our knowledge, data assessing patient-centered outcome, based on affected masticatory muscles and facial nerve, after neurosurgical procedures required for brain tumors or vascular pathology, are scarce.

Therefore, the aim of the present study was to develop and validate a questionnaire for indicating directly affected masticatory muscles groups of facial nerve branches, in patients undergoing the pterional approach in neurosurgery, so that recovery therapy can be monitored and personalized.

## 2. Materials and Methods

### 2.1. Patient Population

This questionnaire-based study was conducted between March 2020 and June 2021, in accordance with ethical principles including the World Medical Association Declaration of Helsinki, the Belmont report, the Council for International Organizations of Medical Sciences (CIOMS) guidelines, and the International Conference on Harmonization in Good Clinical Practice (ICH-GCP). The study was approved by the Bioethical Committee of Polisano Sibiu (877/2020), and the written consent was also obtained for each subject.

Thirty-two consecutive patients having specific conditions that required emergency neurosurgery, being thus operated on in the neurosurgery department at the Polisano-Medlife Clinic, were enrolled in the present study.

The inclusion and exclusion criteria were as follows:

Inclusion criteria: age over 18 years old, surgically treated patients through pterional approach, patients suffering from vascular or tumoral pathology in good mental status, available at the time of evaluation and willing to respond to a questionnaire, upon request.

Exclusion criteria: patients with speech problems, preoperative TMJ pathology, altered general condition, and requirement of adjuvant postoperative radiotherapy/chemotherapy.

### 2.2. Surgical Technique

Surgery was carried out according to standardized surgical and anesthetic techniques. Pterional craniotomy was performed with the patient in a supine position with their head turned to the opposite side and fixed in a Mayfield clamp. Limited shaving of the scalp was performed only for the incision area. The incision was located posterior to the hair insertion line, starting from the midline or the point where the hair insertion line begins (“widow’s peak”), with a curved path downward up to 1 cm anterior to the tragus and 1 cm superior to the zygomatic arch [8].

Damage to the superficial temporal artery should be avoided as it can be important in the course of surgical interventions for vascular pathology, and is used in exo-endocranial bypass. The scalp can be detached from the bone in a single layer, together with the temporalis muscle or the dissection can be performed in a two-layer fashion, the scalp and then the temporalis.

Muscle dissection is performed, in either a posteroanterior or anteroposterior manner. In the posteroanterior variant, the posterior incision of the temporal fascia was achieved and the subperiosteal temporalis muscle was detached, using electrocautery or a subperiosteal elevator. However, electrocautery is not recommended, as it may be involved increasing the postoperative incidence of temporalis muscle atrophy. The method ensuring the most effective preservation of the frontal ramus of the facial nerve was preferred.

For an effective approach, after detaching the temporalis muscle from the bone, the root of the zygomatic arch, as well as the pterion groove, should be visible, and are located anterior and inferior to the frontal-zygomatic suture. Thus, the calvarium is exposed and a craniotomy is carried out, using one or more burr holes.

After performing the surgery, the bone was fastened with plates and screws or other devices (Craniofix, Aesculap AG, Tuttlingen, Germany) to ensure optimal fixation. Muscle closure is important to maintain normal temporalis muscle functionality. Thus, the muscle was sutured using separate absorbable sutures (Vicryl, Ethycon, Lidingö, Sweden), the temporal fascia was also sutured using separate absorbable threads, and scalp closure was achieved using a bi-layered technique entailing the galea aponeurotica and the skin.

### 2.3. Questionnaire

For assessing patient outcomes after surgery, regarding masticatory function and facial nerve involvement, a questionnaire was developed to specifically evaluate affected masticatory muscles groups (Part I) and facial nerve branches (Part II). The questionnaire consists of 18 items: 12 items for rating the degree of damage to the muscles of mastication, the muscles responsible for mandibular movements and TMJ—Table 1, and 6 items regarding the facial nerve involvement—Table 2.

The questionnaires were designed and validated following the three steps: items development, scale development, and scale evaluation, adapted from the recommendations of Boateng et al. [9]. The Romanian language version of the questionnaire is presented in the Supplementary Materials.

#### 2.3.1. Items Development

Each question was developed based on each masticatory muscle function, for Part I (Table 1) and facial nerve motor branches distribution and functions, for Part II (Table 2), using Delphi method.

Immediately upon item design, the questions were pre-tested. Fifteen patients, fulfilling the inclusion criteria, were firstly asked to verify if questions were clear, understandable, and in a logical order.

All items, grouped in masticatory assessment and facial nerve branches integrity assessment, were sent to six medical professionals (three neurosurgeons, one oral and maxillofacial surgeon, one prosthodontist, and one general dentist) to evaluate and criticize the content of the questionnaires.

#### 2.3.2. Scale Development

The adjusted questionnaires, according to patient’s comments and medical professionals’ observations, were applied to fifteen patients enrolled in the present study, for whom the postoperative status was well known from the patients’ medical records and clinical follow-up, in a test–retest fashion, the second interview being one month after the first testing and the intraclass correlation coefficient (ICC) was used to assess reliability.

Construct validation was determined after patient evaluation by a team composed from a neurosurgeon specialist and an oral and maxillofacial surgeon. For validity evaluation it is necessary to prove that the response to the applied items from the questionnaires are correlated with the postoperative mastication dysfunction and facial nerve involvement. Cohen’s coefficient kappa was used to assess expert’s agreement on questions.

The results showed a satisfactory quality of the questionnaires to assess the postoperative outcome of the groups and subgroups of muscles directly involved in the movements of the mandible that are located in the area affected by the surgical intervention as well as possible transitory or permanent palsy of the facial nerve.

#### 2.3.3. Scale Evaluation

Finally, the validated questionnaires were administrated to all the enrolled patients, over the telephone call. The clinical status evaluated by the clinical professionals based on patient’s files, was also recorded. One year after the first interview, the patients were retested to evaluate postoperative recovery in a timeline fashion. No additional recovery treatment was done for patients included in the statistical analysis.

Most of the items are scored with 0 (Yes) and 1 (No), except for item 9, QI.9, (where 1 month is scored 2, 3 months is scored 1 and unable to chew hard food is scored 0) and item 10, QI.10, (scored 0-8) where each subgroup of the question is individually scored 1 and the item’s response is the sum of all subgroups (Table 1). For the question QI.10, “Please chose from the list what food could you chew without having pain on the affected side: (1) banana, kiwi; (2) core bread, soft pizza; (3) liver; (4) gizzard, heart meat; (5) food sticks and pretzels; (6) roasted pork, rabbit, chicken meat; (7) roasted beef; (8) carrots, celery, apple, radishes”, the type of food classification was done as follows: soft (1 + 2); soft and medium (1 + 2 + 3 + 4); medium (1 + 2 + 3 + 4 + 5); medium and hard (1 + 2 + 3 + 4 + 5 + 6 + 7); and hard (1 + 2 + 3 + 4 + 5 + 6 + 7 + 8). The maximum score for the questionnaire (Part I and Part II) is 26, a higher score meaning a better postoperative outcome.

Data on age, gender, time elapsed from the surgery, were also collected.

### 2.4. Statistical Analysis

All data were synthetized in Excel tables, and then compared and analyzed. Statistical analysis was executed with IBM^®^ SPSS^®^ Statistics 25.0 (Armonk, NY, USA). Intraclass correlation coefficient (ICC) was used to assess reliability of the initial questionnaire and Cohen’s coefficient kappa was used for validity evaluation. Pearson chi-square test was used to assess correlations between age, gender, time elapsed from surgery, initial pathology and the response to the questionnaires. Significance was set at a *p*-value < 0.05.

The estimating probability of each dichotomous dependent variable (QI.1 to 8, QI.11, QI.12 and QII.1 to 6) in relationship to the independent variables analyzed age, gender, pathology, time elapsed from the surgery was done using logistic regression. For predictive analytics and modeling, QI.9 and QI.10 were analyzed together (as QI.9 and 10). Only “Yes (I am able to chew hard food)” and “No (I am unable to chew hard food)” options were considered in relation to the same independent variables for estimating probabilities using a logistic regression equation.

For power analysis, G * Power (Version 3.1.9.7 software, Heinrich-Heine University, Dusseldorf, Germany) open software was used. Based on preliminary results from the fifteen pre-tested patients, for a probability of Type 1 error (α) 0.05, an effect size w 0.5 for a total sample of 32 patients, the computed power (1−β) was 0.807.

## 3. Results

**Figure 1:**
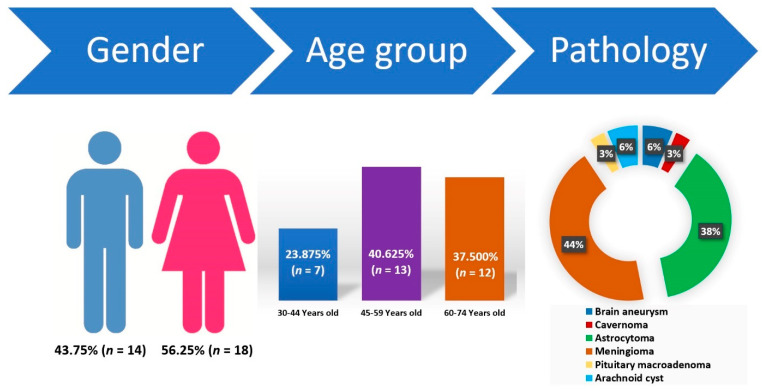
Demographic characteristics and indications for neurosurgery for the enrolled patients: female to male ratio, age distribution of the participants, and distribution of the pathologies.

Among the thirty-two enrolled patients, 56.25% (*n =* 18) were females and 43.75% (*n =* 14) were males, with an age range between 30 and 74 years old, mean 53.94 (±12.68). The pathology was divided as follows: cerebrovascular lesions—9.375% (brain aneurysm—*n* = 2 patients, cavernoma—*n* = 1) and cerebral tumors—90.625% (astrocytoma—*n* = 12, meningioma—*n* = 14, pituitary macroadenoma—*n* = 1, arachnoid cyst—*n* = 2) (Figure 1).

All surgical procedures were performed by the same senior neurosurgeon (C.M.) using the pterional craniotomy approach, as described in Section 2.2. 

No postoperative complications occurred during the assessment period.

Patients in the study group have an almost equal representation between genders with a slight dominance of the female sex. The pathology shows the percentage dominance of meningiomas, followed by astrocytomas which, by age groups, are more common in the age range of 40–50 years.

### 3.1. Devloppment and Validation of the Questionnaires

The initial questionnaire comprised 24 items (14 for Part I and 10 for Part II) and was reduced to 18 items (12 for Part I and 6 for Part II) using the Delphi methodology [10]. The 18 items were applied to fifteen patients fulfilling inclusion criteria, in a test–retest fashion and the ICC values obtained for each items were: 0.720 (Q.I. 1), 0.873 (Q.I. 5, Q.I. 6, Q.I. 7, Q.I. 8) and 1 for the other items, meaning good and excellent reliability [11].

The validation was done on the same group of fifteen patient by two experts (neurosurgeon and oral and maxillofacial surgeon) based on the clinical examinations and patient’s files. The Cohen’s coefficient kappa was 0.815, and was considered excellent by Landys and Koch [12].

### 3.2. Administration of Validated Questionnaire

The validated questionnaire was applied to the thirty-two enrolled patients twice, at a one-year interval (T0 and T1). The time elapsed from surgery was grouped in two main groups, 1 to 5 months, 18.8% (*n* = 6) and 6 to 12 months post-surgery, 81.2% (*n* = 26). Table 3 shows the p values of the response to the questionnaires related to age, gender, time elapsed from surgery and pathology at T0. As can be observed, no statistically significant correlations could be found between postoperative outcomes and age or gender. Facial nerve branch involvement could not be correlated with any of the assessed variables. Pathology was correlated only to QI.4, and time elapsed from surgery was statistically significantly correlated to QI.1, 10–12 (Table 3).

Logistic regression was performed to assess the impact of the independent variables of age, gender, time elapsed from surgery and pathology on the likelihood that the enrolled patients would respond “Yes” to the questions.

The Omnibus Test of Model Coefficient for testing whether the explained variance of data was significantly greater than the unexplained variance; p was 0.77 (QI.1), 0.51 (QI.3), 0.35 (QI.4), 0.12 (QI.5), 0.88 (QI.6), 0.85 (QI.7), 0.95 (QI.8), 0.25 (QI.9–10), 0.35 (QI.11), 0.35 (QI.1 2), 0.37 (QII.1), 0.37 (QII.2), 0.40 (QII.3), 0.35 (QII.4), 0.35 (QII.5), 0.35 (QII.6) ≥ 0.05 and 0.27 (QI.2) < 0.05. Except for QI.2, the predictability of the values of the variable could not be explained by a statistical regression model starting from the independent variables.

For QI.2 (“Do you have pain, on the operated side, while slightly opening your mouth or biting on apples, pears, carrots, or biscuits?”) the following logit regression equation was obtained:

$$\begin{array}{cc} Logit\;\left(QI.2\right)= & 18.555+0.032*\;Age-53.992\;*\;Gender+36.252\\ & *\;Time\_elapsed\_surgery-41.018\;*\;Pathology\;\left(1\right)-0.695\\ & *\;Pathology\;\left(2\right)-1.948\;*\;Pathology\;\left(3\right)-35.057\;*\;Pathology\;\left(4\right)\\ & +15.615\;*\;Pathology\;\left(5\right); \end{array}$$

were Pathology (1) = cavernoma; (2) = astrocytoma; (3) = meningioma; (4) = pituitary macroadenoma; (5) = arachnoid cyst.

**Figure 2:**
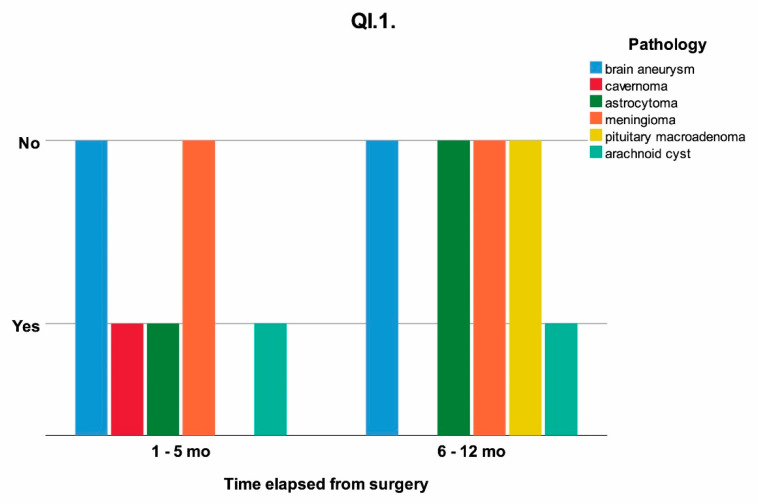
The response at QI.1 (“Do you feel pain in your temple or in the anterior region of the ear when you yawn?”) influenced by the time elapsed from surgery clustered on the initial pathology; mo = months.

**Figure 3:**
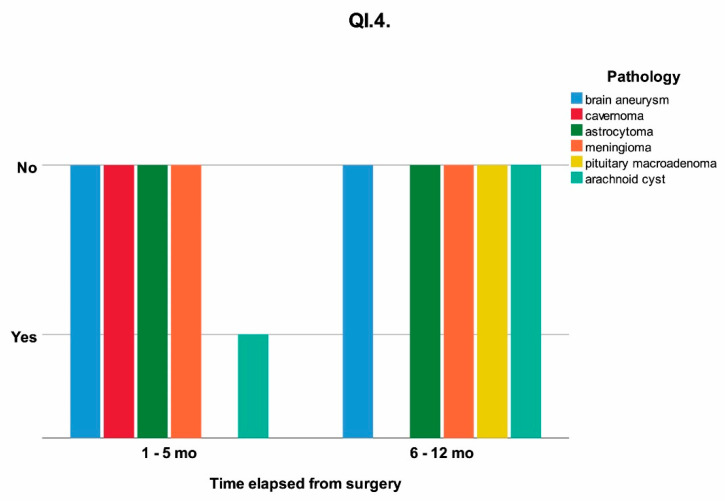
The response at QI.4 (“Did you experience, during/after the healing period, any pain in the anterior region of the ear?”) influenced by the time elapsed from surgery clustered on the initial pathology; mo = months.

**Figure 4:**
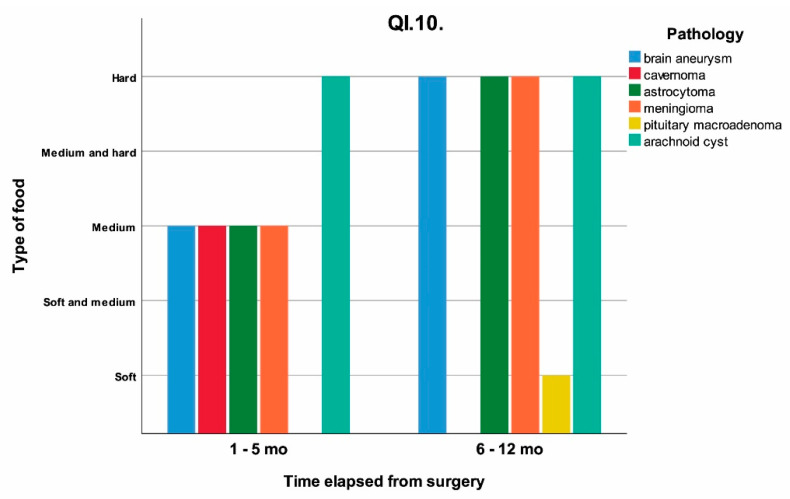
The response at QI.10 (“Please choose from the list what food could you chew without having pain on the affected side”) influenced by the time elapsed from surgery clustered on the initial pathology mo = months.

The influence of time elapsed from surgery on the temporalis muscle, capsular ligaments of the TMJ and types of food chewed without pain were analyzed. The Yes/No answers to questionnaires QI.1 and QI.4, and the type of food able to be chewed without pain (grouped as soft, soft and medium, medium, medium and hard, hard) QI.10 are shown as a timeline, clustered on pathology in Figure 2, Figure 3 and Figure 4.

As can be observed from Table 3 and Figure 2, there is a direct correlation between painful sensitivity during the yawning reflex, which involves all the descending muscles of the mandible, and the time elapsed from surgery, for the studied group, the correlation coefficient was *p* = 0.038 (*p* < 0.05).

For certain pathologies, such as cavernoma, astrocytoma, and arachnoid cyst there was a sensitivity while wide opening of the mouth occurred during the involuntary act caused by yawning, which was not present for the first two pathologies at 6–12 months post-surgery, as can be seen in Figure 2.

Only 3.1% of the patients experienced pain in the anterior region of the ear during and after the healing period, all of them being in 1 to 5 months post surgery. A statistically significant correlation (*p* = 0.008) was also found between the initial pathology and the preauricular pain.

The correlation between the time elapsed after surgery and the restoration of the ability to chew different types of foods is strong, *p* = 0.004 (*p* < 0.05). A total of 37.5% of the patients from the studied group had difficulty in chewing hard food in the first 12 months after neurosurgery (Table 3 and Figure 4). Most of the patients able to chew hard food without pain were in the 6–12 months group.

**Figure 5:**
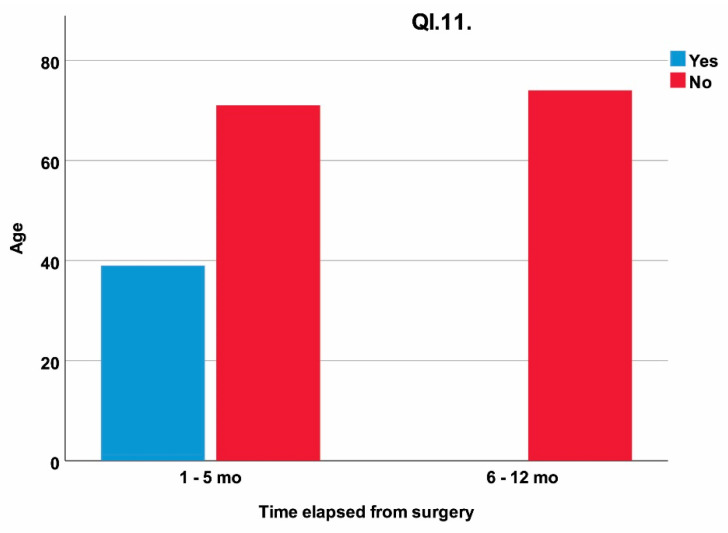
The influence of patient’s age and time elapsed from surgery on the response at QI.11 (“Do you feel pain when moving your lower jaw back and forward, without opening your mouth?”); mo = months.

The influence of age and time elapsed from surgery on mandibular propulsion and retropulsion is illustrated in Figure 5.

The propulsion and retropulsion that intensely involve lateral pterygoid and medial pterygoid in bilateral contraction show a statistically significant correlation to the time elapsed from surgery (*p* = 0.034). A total of 3.1% of patients presented high sensitivity to such movements on the operated side, all patients being 1 to 5 months post surgery.

**Figure 6:**
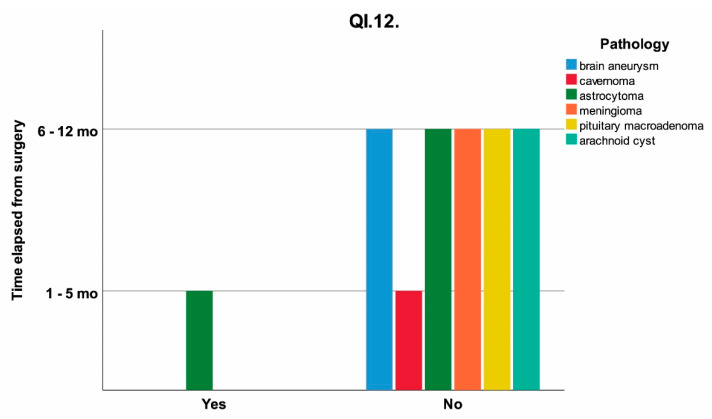
The influence of time elapsed from surgery on the response at QI.12 (“If you open wide and then gradually close your mouth, do you feel pain on the affected side?”) clustered on pathology; mo = months.

The pain when gradually opening the mouth (QI.12), depending on time elapsed from surgery at T0 and pathology is shown in Figure 6.

As presented in Table 3 and Figure 6, there is a statistically significant correlation (*p* = 0.034) between the time elapsed from surgery and gradual mouth closing, movement involving the masseter and temporalis muscle (anterior fibers) by its insertion in the coronoid and lateral pterygoid due to simultaneous contraction. It can be observed that after a period of more than 6 months to 1 year, the impairment of the above-mentioned muscle groups involved in mouth-closing movements were significantly diminished.

As can be seen in Figure 2, Figure 3, Figure 4, Figure 5 and Figure 6, time elapsed from the surgical procedure to the first interview (T0) influenced the overall outcome, an improved functionality was observed in patients interviewed between 6 and 12 months postoperatively.

All patients were reinterviewed one year after the first assessment and a slight increase of the mean total score was noticed (Table 4).

However, at one year after the initial interview, only a few improvements in the range of motion disfunction were noticed. Pain with the yawing reflex diminished but still persisted in 25% of patients, which indicated that the anterior fibers of the temporalis muscle remained functionally affected postoperatively, as well as those of the lateral pterygoid muscle.

Pain while chewing hard food was still present in 25% of patients, with a slightly increasing tendency, as could be observed at recall comparing to the initial interview (21.9%), due probably to temporalis muscle fiber atrophy in the absence of specialized active physiotherapy.

As could be observed by analyzing the response to question QI3 associated with QI4, painful sensitivities to laterality movements on the operated side remained unchanged (12.5% of patients), while pain in the contralateral TMJ increased slightly (in 6.3% compared to 3.1% of patients) showing long-term repercussions on the contralateral TMJ due to the effort in compensating for masticatory function on the affected side.

Pain on contralateral side during the masticatory process (QI.5) was still present in 28.1% of patients, remaining unchanged one year after the first interview.

An inequality between dental arches due to functional shortening of the temporalis muscle fibers (QI.6) remained unchanged at the second follow-up for 12.5% of patients. However, painful closing disappeared at some patients at the second interview, being experienced by 6.3% of patients, comparing to 25% who responded “Yes” to QI.7 at the first interview (Table 4).

The answer to QI.9 did not change obviously, and patients seemed to remember when they were first able to chew hard food.

The response to QI.10 showed that there were no major changes in the consistency of food that patients were able to chew. A slight improvement with chewing medium consistency foods: (1) banana, kiwi; (2) core bread, soft pizza; (3) liver; (4) gizzard, heart meat; and (5) food sticks and pretzels, was noticed in the second interview. A total of 18.8% of patients versus 14.4% were able to chew medium food on a regular basis, without effort or pain.

The back and forward movements involving temporalis muscle and also masseter and pterygoid fibers registered no improvements. A total of 3.1% of patients responded “Yes” to QI.11 in both interviews (Table 4).

Pain with gradual mouth opening (QI.12) was not registered in the second interview, compared to the first one when the answer was “Yes” for 3.1% of patients (Table 4).

Most neurological involvement of the facial nerve and branches remained unchanged at the second interview, as could be noticed from the responded to QII.1–QII.4 (Table 4), all included patients did not have any corrective surgery. Meanwhile, 3.1% of patients with tears flowing over their cheek and an asymmetric smile at the first interview did not complain about these symptoms at the second interview (QII.5 and QII.6, respectively).

## 4. Discussion

The aim of the present study was to develop a self-reporting questionnaire for early diagnosis and assessment of post craniotomy dysfunctions of masticatory muscles and facial nerve branches in neurosurgical patients. The development of the questionnaire emerged due to the need for monitoring, via telephone during the COVID-19 pandemic, the postoperative masticatory status and facial nerve branch involvement of patients undergoing pterional neurosurgical approaches for vascular or tumoral pathologies. The questionnaire was conceived to assess, in a targeted and standardized matter, based on a patient’s feelings, each muscle group on the surgical or contralateral side in order to prescribe, based on a telephone assessment, pain medication, physical therapy, or specialized rehabilitation devices, such as a RehaBite^®^ Bite Pad Trainer, if needed.

The use of telemedicine significantly increased during the COVID-19 pandemic, which during a lock down period is the only means of contacting, monitoring, and advising patients with chronic diseases or those in postoperative care [13,14,15]. The need of maintaining social distancing to reduce infection risks, while also avoiding deterioration to patients’ medical conditions and providing them with an adequate healthcare was the main reason to develop a standardized questionnaire, destined for patient undergoing pterional and other neurosurgical approaches, requiring an incision of the temporal muscle, for different cranial pathologies.

Dissection of the temporal muscle during craniotomy is frequently associated with its postoperative atrophy, restricted mandibular movements and the development of signs and symptoms of TMJ dysfunction [16]. In addition to functional disability, cosmetic defects could also result from the dissection of the frontal branch of the facial nerve, such as paralysis of the forehead muscle, the orbicularis oculi muscle, and the corrugator supercilii muscle [17], all these aspects can cause severe impairment of a patient’s quality of life [18].

Most of the above-mentioned complications could be minimized when diagnosed early and an adequate treatment prescribed.

Patient-centered outcomes assessment questionnaires are objective and widely accepted measurement tools for the assessment of health care quality and are essential for providing patient-centered care [19,20,21,22]; they are strongly recommended by the European Medicines Agency and the U.S. Food and Drug Administration as relevant endpoints for approving new therapeutical strategies [7].

According to our knowledge, there are no specifically designed questionnaires for the evaluation of patients with neurosurgical interventions requiring an incision of temporalis muscles and facial nerve branch dissections.

Abdulazim et al. [6] proposed five items for the evaluation of postcraniotomy complications and complaints according to temporal muscle dysfunction. The five items comprised patients’ complaints associated with the surgery; time after surgery when complains were registered; whether these complaints led to other medical advice or intervention; if a relief occurred spontaneously or after medical intervention; and if preexisting morbidity or disorders were present [6].

Gierthmuehlen et al. [23] used the German version of Oral Health Impact Profile (OHIP) 14 to evaluate the oral health-related quality of life of patients who were operated through a fronto-lateral-pterional, pterional, or temporal approach for neurosurgical pathologies.

For oncological patients, the European Organization for Research and Treatment of Cancer (EORTC) developed modular questionnaires assessing the quality of life of cancer patients, with EORTC QLQ-C30 as the core, specific to the tumor site and treatment modality, which is extremely useful in clinical cancer trials. Guidelines the development of other modules were also provided by EORTC [24].

Different to the above-mentioned questionnaire, which aimed to evaluate the quality of life, our proposed questionnaire was designed to directly evaluate each muscle group or the involved facial nerve branches, based on patients’ self-reported postoperative outcomes, in a comprehensive way, to be able to specifically address patient complains. Each specific question corresponded to a masticatory muscle or specific facial nerve branches, as noted in Table 1 and Table 2. The design of the present subjective assessment tool was tailored to evaluate the side-effects of neurosurgery on the masticatory muscles, TMJ function of the affected and contralateral side and facial nerve branches involved, to be able to prevent and treat muscle atrophy, as well as further functional and esthetic consequences, without the need of multiple follow-up visits, which are difficult to accomplish due to the COVID-19 pandemic.

It is well known that, so far, none of the neurosurgical techniques requiring disconnection of the temporalis muscle have managed to entirely prevent its atrophy [25,26]. However, not only is the temporalis muscle affected, its mobilization generates an alteration in normal TMJ function, which manifests as pain, a limited ability to chew, and problems with occlusion, mouth opening, and lateral movement of the jaw [16,18]. These functional alterations were observed in the patients in our study group. For example, pain while chewing or associated with the yawing reflex persisted in 25% of the patients, even after more than one year post surgery (at the second interview). Additionally, 37.5% of the included patients avoided hard food, with no improvements observed by the second interview (Table 4).

Several studies pointed out the fact that patients undergoing the same surgical procedure for craniotomy may or may not develop muscle atrophy [27]. In a study on 24 patients with pre-temporal craniotomy for refractory mesial temporal lobe epilepsy, Costa et al. highlighted the association of pre-operative bruxism with an increased risk of post craniotomy temporomandibular disorder [16].

There is a connection between the neurosurgical approach and the anatomical structures sharing the same territory of interest, and the connection becomes evident in the immediate postoperative period and throughout the recovery period. Pathology and time elapsed from surgery were statistically significantly correlated to preauricular pain on the non-operated side (*p* = 0.008 and *p* = 0.034, respectively), due probably to the effort made by the contralateral TMJ to compensate for masticatory movements and temporalis muscle atrophy on the operated side.

A correlation between time elapsed from surgery and pain while yawning, pre-auricular pain, the ability to chew hard food, preauricular pain during lateral jaw movements, and gradually mouth opening, was noticed by our study group (Table 3). Similar results of spontaneous relief of painful symptoms and different functional outcomes were reported by Kawaguchi et al. [5].

The goal of our proposed questionnaire was to highlight postoperative recovery issues in order to obtain preliminary data regarding the effectiveness of this neurosurgical technique, which we consider, according to the results obtained from the assessed patients, to have high potential for postoperative recovery.

The postoperative protocol agreed to in our neurosurgery department requires clinical and imagistic reassessment at least one year after surgery. Magnetic resonance imaging (MRI) was used for paraclinical reevaluation and clinically, a neurologist assessed the patients, respecting the epidemiological norms imposed by the pandemic. In addition, between the neurosurgical monitoring, a follow-up from a dental specialist or oral and maxillofacial surgeon for early mobilization of masticatory muscles, preventing their atrophy, TMJ problems, or facial asymmetry is mandatory, and is also part of the follow-up protocol. Considering the particular situation of social distancing and the lock down of dental practices during the first wave of the COVID-19 pandemic, the telephone questionnaire developed was proved to be useful for patient monitoring, routine follow-up, and as a replacement for recall appointments.

In spite of the reduced number of patients enrolled in our study, the present patient-centered outcome evaluation of postoperative recovery post craniotomy provided an overview of the postoperative course of patient’s complaints regarding masticatory function and facial nerve branch involvement, and whether they disappear after a certain amount of time.

The present study has some limits. One of the limits of the present study was the fact that patients were not examined by a dentist or oral and maxillofacial surgeon before neurosurgery, so a preoperative evaluation of masticatory status was not possible.

In addition, an assessment of quality of life using an OHIP-validated questionnaire was not administrated in association to the present questionnaire.

The developed questionnaire could be a useful tool for evaluating different surgical techniques, and also objectively monitors the efficiency of physical therapy, or specialized rehabilitation devices in improving masticatory and aesthetic functions.

## 5. Conclusions

We created and validated a valuable patient-centered questionnaire that can be employed as a tool for postoperative assessment of directly affected masticatory muscle groups of facial nerve branches. The proposed questionnaire could be used for routine follow-ups regarding improving masticatory and aesthetic functions, between the neurological recall appointments.

## Figures and Tables

**Table 1:** Questionnaire Part I for the evaluation of the affected masticatory muscles groups and the temporomandibular joint (TMJ).

Part I	When Thinking About Last Month, Please Answer the Following Questions:	Answer Options	Involved Muscle Group and TMJ
1	Do your feel pain in your temple or in the anterior region of the ear, on the operated side, when you yawn?	Yes/No	Temporalis muscle posterior fibers, masseter muscle, lateral pterygoid muscle—affected side
2	Do you have pain, on the operated side, while slightly opening your mouth or biting apples, pears, carrots, or biscuits?	Yes/No	Temporalis muscle anterior fibers, lateral pterygoid muscle—affected side
3	When moving your jaw laterally, to the operated side, do you feel pain in your temple or in the anterior region of the ear?	Yes/No	Lateral pterygoid muscle from the opposite side, medial pterygoid muscle affected side; capsular ligaments of the TMJ—affected side
4	Did you experience any pain in the anterior region of the ear on the non-operated side?	Yes/No	Capsular ligaments of the TMJ—opposite side
5	When biting on the opposite side, do you feel any pain in the operated side?	Yes/No	Lateral pterygoid muscle, superior head from the affected side medial pterygoid muscle on the opposite side
6	When you bite slightly on the posterior teeth, do you feel the teeth touching each other in different way on normal and operated side?	Yes/No	Volumetric asymmetry of the temporalis muscles
7	If your answer was Yes to the previous question please specify if bringing teeth together on the operated side is painful.	Yes/No	Temporalis muscle posterior fibers, masseter muscle on the affected side
8	When swallowing chewed food, do you feel any pain on the operated side?	Yes/No	Temporalis muscle posterior fibers
9	At how long after surgery were you able to chew hard food?	1 month/3 month/I am unable to chew hard food at this time	Temporalis muscle anterior fibers, lateral pterygoid muscle—affected side
10	Please choose from the list what foods you could chew without having pain on the operated side:	(1) banana, kiwi; (2) core bread, soft pizza; (3) liver; (4) gizzard, heart meat; (5) food sticks and pretzels; (6) roasted pork, rabbit, chicken meat; (7) roasted beef; (8) carrots, celery, apple, radishes	Masseter, temporalis, medial and lateral pterygoid muscles
11	Do you feel pain when moving your lower jaw back and forward, without opening your mouth?	Yes/No	Temporalis muscle anterior fibers, lateral pterygoid muscles
12	If you open wide and then gradually close your mouth, do you feel pain on the operated side?	Yes/No	Temporalis muscle anterior fibers and the suprahyoid muscle group (digastric, mylohyoid, and geniohyoid muscles)—they depress the mandible/temporalis muscle posterior fibers and elevator muscles (masseter, lateral and medial pterygoid muscles)

Each item is scored Yes = 0; No = 1; for item 9, 1 month is scored 2, 3 months is scored 1 and unable to chew hard food is scored 0; for item 10 each subgroup is scored 1 and the item’s response is the sum of all subgroups.

**Table 2:** Questionnaire Part II for facial nerve branches integrity assessment.

Part II	When Thinking about Last Month, Please Answer the Following Questions:	Answer Options	Involved Facial Nerve Branches
1	Did you experience difficulties in complete eye closure on the operated side?	Yes/No	Frontal branch of the facial nerve
2	Did you notice asymmetric forehead wrinkles (fewer wrinkles on the operated side)?	Yes/No	Frontal branch of the facial nerve
3	Did you notice difficulties in forceful eye closure?	Yes/No	Zygomatic branch of the facial nerve
4	Did you experience speech impairments, difficulty while smiling, moving your mouth or unilaterally flattening of the nasolabial fold with facial asymmetry?	Yes/No	Buccal branch of the facial nerve
5	Did you notice tears flowing over your cheek on the operated side?	Yes/No	Frontal branch of the facial nerve
6	Did you notice asymmetric smile and problems with eating or drinking?	Yes/No	Marginal mandibular branch of the facial nerve

Each item is scored Yes = 0; No = 1.

**Table 3:** Pearson chi-square test with *p* values at T0.

	Age	Gender	Time Elapsed from Surgery	Pathology
QI.1	0.320	0.773	0.038 *	0.064
QI.2	0.284	0.075	0.064	0.355
QI.3	0.352	0.788	0.087	0.142
QI.4	0.979	0.37	0.034 *	0.008 *
QI.5	0.348	0.96	0.753	0.062
QI.6	0.157	0.419	0.304	0.955
QI.7	0.493	0.681	0.601	0.771
QI.8	0.798	0.37	0.625	0.932
QI.9	0.562	0.244	0.185	0.859
QI.10	0.447	0.033	0.004 *	0.143
QI.11	0.798	0.37	0.034 *	0.886
QI.12	0.798	0.37	0.034 *	0.886
QII.1	0.725	0.178	0.304	0.593
QII.2	0.725	0.178	0.304	0.593
QII.3	0.929	0.401	0.382	0.494
QII.4	0.979	0.249	0.625	0.886
QII.5	0.798	0.37	0.625	0.932
QII.6	0.798	0.37	0.625	0.932

(*) and bold = statistically significant *p* < 0.05.

**Table 4:** Response to questionnaire (19 items) at T0 and T1.

Question	Answer T0	Answer T1
QI.1	31.3% (Yes)	25.0% (Yes)
QI.2	21.9% (Yes)	25.0% (Yes)
QI.3	12.5% (Yes)	12.5% (Yes)
QI.4	3.1% (Yes)	6.3% (Yes)
QI.5	28.1% (Yes)	28.1% (Yes)
QI.6	12.5% (Yes)	12.5% (Yes)
QI.7	25.0% (Yes)	6.3% (Yes)
QI.8	3.1% (Yes)	0% (Yes)
QI.9	84.4% (at 1 month)	84.4% (at 1 month)
	15.6% (at 3 month)	15.6% (at 3 month)
	0% (unable)	0% (unable)
QI.10	6.3% (soft food)	6.3% (soft food)
	12.5% (soft and medium food)	9.4% (soft and medium food)
	15.6% (medium food)	18.8% (medium food)
	3.1% (medium and hard food)	3.1% (medium and hard food)
	62.5% (hard food)	62.5% (hard food)
QI.11	3.1% (Yes)	3.1% (Yes)
QI.12	3.1% (Yes)	0% (Yes)
QII.1	12.5% (Yes)	12.5% (Yes)
QII.2	12.5% (Yes)	12.5% (Yes)
QII.3	9.4% (Yes)	9.4% (Yes)
QII.4	3.1% (Yes)	3.1% (Yes)
QII.5	3.1% (Yes)	0% (Yes)
QII.6	3.1% (Yes)	0% (Yes)
Mean total score (±SD)	23.00 (±2.98)	23.34 (±2.82)

## Data Availability

Not applicable.

## Supplementary Materials

The following are available online at https://www.mdpi.com/article/10.3390/jcm11010065/s1, Table S1: Questionnaire Part I for the evaluation of the affected masticatory muscles groups and the temporomandibular joint (TMJ) (Romanian), Table S2: Questionnaire Part II for facial nerve branches integrity assessment (Romanian).

## References

[1] Peeters S., July J., July J., Wahjoepramono E.J. (2019). Pterional Approach BT-Neurovascular Surgery: Surgical Approaches for Neurovascular Diseases.

[2] Yasargil M.G., Antic J., Laciga R., Jain K.K., Hodosh R.M., Smith R.D. (1976). Microsurgical pterional approach to the aneurysms of the basilar bifurcation. Surg. Neurol..

[3] Rocha-Filho P.A.S., Fujarra F.J.C., Gherpelli J.L.D., Rabello G.D., de Siqueira J.T.T. (2007). The long-term effect of craniotomy on temporalis muscle function. Oral Surg. Oral Med. Oral Pathol. Oral Radiol..

[4] de Andrade Júnior F.C., de Andrade F.C., Araujo Filho C., Carcagnolo Filho J. (1998). Dysfunction of the temporalis muscle after pterional craniotomy for intracranial aneurysms: Comparative, prospective and randomized study of one flap versus two flaps dieresis. Arq. Neuropsiquiatr..

[5] Kawaguchi M., Sakamoto T., Furuya H., Ohnishi H., Karasawa J. (1996). Pseudoankylosis of the mandible after supratentorial craniotomy. Anesth. Analg..

[6] Abdulazim A., Filis A., Sadr-Eshkevari P., Schulte F., Sandu N., Schaller B. (2012). Postcraniotomy function of the temporal muscle in skull base surgery: Technical note based on a preliminary study. Sci. World J..

[7] De Cicco D., Tartaro G., Ciardiello F., Fasano M., Rauso R., Fiore F., Spuntarelli C., Troiano A., Lo Giudice G., Colella G. (2021). Health-Related Quality of Life in Oral Cancer Patients: Scoping Review and Critical Appraisal of Investigated Determinants. Cancers.

[8] Rubio R.R., Chae R., Vigo V., Abla A.A., McDermott M. (2019). Immersive surgical anatomy of the pterional approach. Cureus.

[9] Boateng G.O., Neilands T.B., Frongillo E.A., Melgar-Quiñonez H.R., Young S.L. (2018). Best Practices for Developing and Validating Scales for Health, Social, and Behavioral Research: A Primer. Front. Public Health.

[10] Hasson F., Keeney S., McKenna H. (2000). Research guidelines for the Delphi survey technique. J. Adv. Nurs..

[11] Koo T.K., Li M.Y. (2016). A Guideline of Selecting and Reporting Intraclass Correlation Coefficients for Reliability Research. J. Chiropr. Med..

[12] Landis J.R., Koch G.G. (1977). The Measurement of Observer Agreement for Categorical Data. Biometrics.

[13] Schiller T., Zornitzki T., Ostrovsky V., Sapojnik D., Cohen L., Kunyavski T., Knobler H., Kirzhner A. (2021). Following the COVID-19 Experience, Many Patients with Type 1 Diabetes Wish to Use Telemedicine in a Hybrid Format. Int. J. Environ. Res. Public Health.

[14] Medar C., Cristache C.M., Mihut T., Marcov E.C., Furtunescu F.L., Burlibasa M., Burlibasa L. (2020). Defensive dentistry from normal medical practice to safeguard from malpractice litigations. New rules in COVID-19 pandemic. Rom. J. Leg. Med..

[15] Dopelt K., Avni N., Haimov-Sadikov Y., Golan I., Davidovitch N. (2021). Telemedicine and eHealth Literacy in the Era of COVID-19: A Cross-Sectional Study in a Peripheral Clinic in Israel. Int. J. Environ. Res. Public Health.

[16] Costa A.L.F., Yasuda C.L., França M., de Freitas C.F., Tedeschi H., de Oliveira E., Cendes F. (2014). Temporomandibular dysfunction post-craniotomy: Evaluation between pre-and post-operative status. J. Cranio-Maxillofac. Surg..

[17] Matsumoto K., Akagi K., Abekura M., Ohkawa M., Tasaki O., Tomishima T. (2001). Cosmetic and functional reconstruction achieved using a split myofascial bone flap for pterional craniotomy. J. Neurosurg..

[18] Valdivia-Chiñas H., Córdoba-Mosqueda M.E., Cruz-Cruz E.F., Ochoa-Cacique D., Medina-Carrillo Ó., García-González U. (2019). Evaluation of temporal muscle trophism in relation to the manipulation time and infiltration of 0.5% isobaric bupivacaine through a pterional approach. Neurocirugía.

[19] Rudolph C., Petersen G.S., Pritzkuleit R., Storm H., Katalinic A. (2019). The acceptance and applicability of a patient-reported experience measurement tool in oncological care: A descriptive feasibility study in northern Germany. BMC Health Serv. Res..

[20] Cristache C.M., Totu E.E., Iorgulescu G., Pantazi A., Dorobantu D., Nechifor A.C., Isildak I., Burlibasa M., Nechifor G., Enachescu M. (2020). Eighteen Months Follow-Up with Patient-Centered Outcomes Assessment of Complete Dentures Manufactured Using a Hybrid Nanocomposite and Additive CAD/CAM Protocol. J. Clin. Med..

[21] Constantin A.M., Lupusoru M.O.-D. (2015). Socio-demographic and economic characteristics of patients with psychiatric pathology and non-fatal suicidal behavior. Rom. J. Leg. Med..

[22] Cristache C.M., Ionescu C., Burlibaşa M., Cristache G., Iliescu A.A., Dumitriu H.T. (2009). Retentive anchors versus magnets as attachment systems for mandibular overdenture. A 5-year prospective randomised clinical study. Metal. Int..

[23] Gierthmuehlen M., Jarc N., Plachta D.T.T., Schmoor C., Scheiwe C., Gierthmuehlen P.C. (2021). Mastication after craniotomy: Pilot assessment of postoperative oral health-related quality of life. Acta Neurochir..

[24] European Organisation for Research and Treatment of Cancer (EORTC). https://www.eortc.org/.

[25] Park J.-H., Lee Y.-S., Suh S.-J., Lee J.-H., Ryu K.-Y., Kang D.-G. (2015). A simple method for reconstruction of the temporalis muscle using contourable strut plate after pterional craniotomy: Introduction of the surgical techniques and analysis of its efficacy. J. Cerebrovasc. Endovasc. Neurosurg..

[26] Welling L.C., Figueiredo E.G., Wen H.T., Gomes M.Q.T., Bor-Seng-Shu E., Casarolli C., Guirado V.M.P., Teixeira M.J. (2015). Prospective randomized study comparing clinical, functional, and aesthetic results of minipterional and classic pterional craniotomies. J. Neurosurg..

[27] Yasuda C.L., Costa A.L., Franca M., Pereira F.R., Tedeschi H., De Oliveira E., Cendes F. (2010). Postcraniotomy temporalis muscle atrophy: A clinical, magnetic resonance imaging volumetry and electromyographic investigation. J. Orofac. Pain.

