# Supplementary material for: Questionnaire-Based Assessment of the Masticatory Function and Facial Nerve Recovery Post Pterional Approach in Brain Tumors Surgery
Source: J Clin Med. 2021 Dec 23;11(1):65. doi: 10.3390/jcm11010065 (PMC8745324; doi:10.3390/jcm11010065)
Supplement: Supplementary file 1 [file jcm-11-00065-s001.zip › jcm-1506495-supplementary.pdf]

Table S1: Questionnaire Part I for the evaluation of the affected masticatory muscles groups and the temporomandibular joint (TMJ) (Romanian)

**Partea I**	**Dacă luăm în considerare ultima lună, vă rugăm să răspundeți la următoarele întrebări:**	**Opțiuni de răspuns**
1	Când căscați vă doare tâmpla sau regiunea anterioară a urechii pe partea operată?	Da/Nu
2	Când deschideți ușor gura sau mușcați dintr-o felie de măr, pară, morcov sau biscuit, doare în zona operată?	Da/Nu
3	Când miscați mandibula în lateral, spre zona operată doare la tâmplă sau în regiunea anterioară a urechii?	Da/Nu
4	Aveți dureri în regiunea anterioară a urechii pe partea neoperată?	Da/Nu
5	Când mestecați pe partea opusă, simțiți durere în partea operată?	Da/Nu
6	Când mușcați ușor pe dinții posteriori, simțiți că dinții se ating diferit față de normal pe partea operată?	Da/Nu
7	Dacă răspunsul este Da la precedenta întrebare, vă rog să specificați dacă simțiti durere atunci când aduceți forțat dinții în contact pe partea operată.	Da/Nu
8	Când înghițiți alimentele mestecate simțiți durere pe partea operată?	Da/Nu
9	La cât timp după intervenția chirurgicală ați putut mesteca alimente dure?	1 lună/3 luni/în prezent sunt incapabil să mestec alimente dure
10	Vă rog să alegeți din listă ce alimente puteți mesteca fără a avea dureri pe partea operată:	1) banană; kiwi; 2) miez de pâine, pizza moale; 3) ficat; 4) pipotă, inimă; 5) sticks-uri și covrigi; 6) friptură de porc, iepure, friptură de pui; 7) friptură de vită; 8) morcovi, țelină, măr, ridiche
11	Simțiți durere când duceți manibula înainte și înapoi fără a deschide gura?	Da/Nu
12	Simțiți durere pe partea operată când deschideți larg gura iar apoi o închideți gradual?	Da/Nu

Scorul fiecărui item este Da=0; Nu=1; pentru item-ul 9, 1 lună=2, 3 luni=1 iar în prezent sunt incapabil să mestec alimente dure=0; Pentru item-ul 10 fiecare subgrup are 1 punct iar scorul total este obținut prin adunarea tuturor punctelor de la fiecare subgrup.

Table S2: Questionnaire Part II for facial nerve branches integrity assessment (Romanian)

**Partea II**	**Dacă luăm în considerare ultima lună, vă rugăm să răspundeți la următoarele întrebări:**	**Opțiuni de răspuns**
1	Ați avut dificultăți în închiderea complete a ochiului de partea operată?	Da/Nu
2	Ați observant o încrețire asimetrică a ridurilor de la nivelul frunții (mai puține riduri pe partea operată)?	Da/Nu
3	Ați observant dificultăți în închiderea forțată a ochiului de partea operată?	Da/Nu
4	Ați avut dificultăți în vorbire, zâmbet sau în mișcările laterale ale gurii? Ați observat absența cutelor nazolabiale cu asimetrie facială?	Da/Nu
5	Ați remarcat lacrimi curgând pe obrazul de partea operată?	Da/Nu
6	Ați remarcat că aveți un zâmbet asimetric sau probleme în timp ce mâncați sau beți lichide?	Da/Nu

Scorul fiecărui item este Da=0; Nu=1.
